# Associations between Internet addiction, life satisfaction, and post-traumatic stress symptoms among disaster-exposed children and early adolescents: a three-wave longitudinal study

**DOI:** 10.3389/fpsyt.2025.1628873

**Published:** 2025-10-24

**Authors:** Wei Shi, Guangzhe Yuan, Zhangming Chen, Youguo Tan, Lihua Jiang, Li Zhao

**Affiliations:** ^1^ Sichuan University–The Hong Kong Polytechnic University Institute for Disaster Management and Reconstruction, Sichuan University, Chengdu, China; ^2^ Department of Psychiatry, NYU Grossman School of Medicine, New York, NY, United States; ^3^ School of Education Science, Leshan Normal University, Leshan, China; ^4^ Zigong Red Cross Society, Zigong, China; ^5^ Zigong Mental Health Center, Zigong, China; ^6^ General Practice Ward/International Medical Center Ward, General Practice Medical Center, West China Hospital, Sichuan University, Chengdu, China; ^7^ Department of health policy and management, West China School of Public Health and West China Fourth Hospital, Sichuan University, Chengdu, China

**Keywords:** Internet addiction, life satisfaction, post-traumatic stress symptoms, children, young adolescents

## Abstract

**Objectives:**

Previous studies have established links between Internet addiction, life satisfaction, and post-traumatic stress symptoms (PTSS) in individuals exposed to disasters; however, the nature of longitudinal links remains uncertain. This study aimed to determine the potential associations among Internet addiction, life satisfaction and PTSS among disaster-exposed Chinese children and early adolescents.

**Methods:**

A three-wave longitudinal design was employed over two years and included 2354 Chinese children and early adolescents aged 6 years to 14 years who directly experienced an earthquake and COVID-19. A complete longitudinal mediation method was applied to analyze data collected in three waves from 2020 to 2022 with a one-year interlude.

**Results:**

Life satisfaction at Time 2 (T2) fully mediated the associations between Internet addiction at Time 1 (T1) and PTSS at Time 3 (T3). Internet addiction at T2 was a significant mediator of links between life satisfaction at T1 and PTSS at T3.

**Conclusions:**

These findings underscore the pivotal role of life satisfaction in mediating the impact of early Internet addiction on long-term psychological outcomes, highlighting it as a key target for interventions aimed at enhancing mental well-being after disaster exposure. Moreover, Internet addiction mediated the link between earlier life satisfaction and later PTSS, highlighting the importance of reducing problematic Internet use to prevent long-term stress symptoms.

## 1 Introduction

In recent years, Internet addiction (IA) has become among the most concerning adverse mental reactions among young people after experiencing stressful and traumatic life events (1). IA refers to a social problem characterized by typical symptoms such as excessive preoccupation with being online, persistent urges to use the Internet, neglect of responsibilities in real life, and intense emotional discomfort with online disconnection (2). Previous research has reported that after experiencing disasters, adolescents and young adults report an increase in IA symptoms [e.g (3–6).,]. Children and adolescents frequently used the Internet for various activities during COVID-19, such as maintaining social connections, engaging in distance learning, doing homework online, and accessing information on diverse topics (4). They are more susceptible to IA because they are in the major developmental period in terms of physiology, psychology, and socialization (1). In addition, the average time spent on the Internet increased during COVID-19, which increased IA risk among children and adolescents exposed to disasters (4). Prior studies found that 13.6% of adolescents experienced IA before the COVID-19 pandemic (7) and 24.4% of adolescents had IA during this period (8). In addition, previous research on an earthquake in Turkey indicated that survivors increased their Internet usage as a coping mechanism for stress, which could easily result in problematic use after experiencing a disaster (9).

Previous research findings indicate that IA occurrence is often accompanied by the development of psychological disorders after a disaster, such as post-traumatic stress symptoms (PTSS) (4). PTSS are defined as an individual’s adverse psychological responses and symptoms (e.g., hyperarousal, flashbacks, emotional numbness, and intrusive thoughts) after a traumatic experience, such as a disaster (10). PTSS were strongly correlated with IA in previous studies (7, 11–13). The studies noted that a more severe level of post-traumatic stress disorder (PTSD) symptoms was significantly linked with more problematic Internet use among 392 South Korean students after their experience with the Sewol ferry disaster (14, 15). Based on a sample of 776 middle-school Chinese students, one study found a strong positive association between IA symptoms and PTSS after the Wenchuan earthquake (5). Additionally, a preceding study, after controlling for age and recurring trauma exposure, reported a significant and positive relationship between PTSD and IA symptoms among 767 post-earthquake adolescents (16). A study based on a sample of 554 survivors exposed to the Turkey earthquake reported that a higher IA level was significantly associated with more severe PTSD symptoms (9).

Multiple longitudinal studies have implied a potential predictive effect of IA on PTSS following traumatic events, especially among young people with sociodemographic vulnerabilities and who have experienced severe disasters. One such study, based on a sample of 7–958 Chinese adolescents exposed to COVID-19, employed network analysis to reveal the major comorbid symptoms between IA and PTSD symptoms, suggesting IA could play a significant role in the persistence of PTSD symptoms over time (4). Another two-month longitudinal study of 811 Chinese adolescents who experienced COVID-19 applied cross-lagged panel network modeling to examine the relationships between PTSD symptoms and IA. The results revealed that high and low severity of IA strongly predicted the hyperarousal symptom from PTSD and that IA could significantly impact progression of PTSD symptoms (17).

Previous studies have suggested that increased IA in the early stages following exposure to stressful events may contribute to more severe long-term PTSS, potentially through the mediating effect of decreased life satisfaction (LS) (18, 19). LS refers to an individual’s overall evaluation of life quality and reflects how positively they generally perceive their life (20). A comprehensive meta-analysis of 18 studies found that individuals with higher IA levels tended to report significantly lower quality of life and poorer psychological well-being (21). Similarly, a global meta-analysis across 13 countries found that a higher national prevalence of IA was significantly associated with lower national levels of LS (22). In addition, a cross-cultural study involving 943 participants reported a significant negative association between PTSD symptoms and LS among young adults (23). Several other studies have also supported this negative relationship between PTSD and LS (e.g., 24, 25). Moreover, some research has suggested that the onset and persistence of IA may lead to inadequate LS (26), which could further impair mental well-being and elevate the risk of developing mental disorders such as PTSD in individuals exposed to various stressful events [e.g (27, 28)].

Several theories and studies suggest that LS may play a mediating role in the longitudinal relationship between IA and PTSS. For example, self-determination theory posits that mental well-being depends on the fulfillment of three basic psychological needs including autonomy (i.e., feeling in control and making self-determined decisions without external pressure), competence (i.e., feeling capable and effective in managing challenges), and relatedness (i.e., feeling connected, supported, and close to others) (29). IA could hinder the satisfaction of these needs, leading to decreased LS and an increased risk of negative psychological outcomes such as PTSS (30). Previous research has shown that excessive Internet use, particularly when characterized by maladaptive patterns such as escapism or social withdrawal, might compromise the fulfillment of these basic psychological needs, thereby resulting in diminished LS (31). Reduced LS may weaken psychological resilience and adaptive coping mechanisms, which could heighten individuals’ vulnerability to trauma-related symptoms (21). Accordingly, LS may serve as a mediating factor in the relationship between IA and PTSS as addictive behaviors disrupt the satisfaction of basic psychological needs, thereby reducing mental well-being and increasing susceptibility to trauma-related symptoms following disaster exposure.

The cognitive-behavioral model (CBM) has also contributed to establishing that LS may mediate the longitudinal relationship between IA and PTSS (32). CBM posits that thoughts, feelings, and behaviors are interrelated and mutually influential (32). IA is frequently characterized by maladaptive cognitive patterns (e.g., beliefs such as “being online is the only way I can unwind”) and avoidance behaviors (e.g., engaging in compulsive Internet use to avoid real-life stressors). These patterns may restrict engagement in meaningful offline activities and social relationships, thereby diminishing overall LS (33). Moreover, as individuals increasingly rely on the Internet as a coping mechanism, they may develop cognitive distortions (e.g., beliefs such as “I am only competent in online contexts”), experience reduced real-world functioning and social connectedness, and begin to neglect personal aspirations and achievements. These experiences can undermine LS, a fundamental dimension of psychological well-being (34). Previous research has indicated that lower LS is associated with reduced psychological resilience, increased vulnerability to trauma-related stress, more negative appraisals of traumatic experiences, and decreased use of adaptive coping strategies (9, 35, 36). These factors may increase individuals’ susceptibility to developing PTSS following trauma exposure induced by disaster events (24). Accordingly, LS could mediate the impact of IA at the onset on prolonged PTSS by impacting the psychological consequence of cognition and behavior adjustment and influencing coping capacity after trauma. Based on the above, we hypothesized that LS at the one-year follow-up would mediate the association between baseline IA and PTSS at the two-year follow-up among disaster-exposed children and early adolescents exposed to the disaster.

Although prior research has highlighted the impact of IA on PTSS, few longitudinal studies have explored the mediating role of LS in their relationship among disaster-exposed children and early adolescents. To address this gap, this study examined the longitudinal associations among IA, LS, and PTSS across two years. Utilizing a three-wave longitudinal design, the study involved a large sample of children and early adolescents affected by disasters. The findings should enhance our understanding of the developmental pathways linking behavioral addiction to trauma-related mental symptoms and to inform targeted interventions that support psychological well-being in post-disaster contexts.

## 2 Materials and methods

### 2.1 Participants and procedure

The study data were obtained from the Chengdu Positive Child Development project, which conducted a three-wave study among Chinese children and adolescents during the COVID-19 lockdown period (37). Chengdu was one of the worst-affected cities in China during COVID-19. Moreover, as the capital of Sichuan province in western central China, Chengdu was strongly impacted by earthquakes of different magnitude levels that occurred in China. All participants in this three-wave study experienced the most challenging and severe phase of the COVID-19 pandemic in Chengdu. While the pandemic was the primary factor, the participants may have also been secondarily affected by earthquakes of different intensities.

A random cluster sampling method was used to select five schools in Chengdu to recruit the project participants, including three high schools and two schools that provide integrated primary and middle education under unified management. A self-administered questionnaire was used to collect data. Three waves of data were collected from 2020 to 2022 with a one-year interval, including baseline (Time 1, T1: June–July 2020), first follow-up (Time 2, T2: June 2021), and second follow-up (Time 3, T3: June 2022). A total of 7–393 participants were enrolled at T1. Of these, 5–913 remained at T2 and 2–354 completed the study at T3, reflecting attrition rates of 20% from T1 to T2 and 60% from T2 to T3. Inconsistent regional lockdowns during the COVID-19 outbreak posed logistical challenges for data collection and contributed to increased participant attrition across time points.

Attrition analyses revealed that participants who dropped out were significantly more likely to be older, exhibited higher baseline IA levels, and reported lower LS (*p*s <.001). No significant differences were observed in sex (*p* = .076) or PTSS (*p* = .828) between those who completed all three waves and those lost to follow-up. The final sample for the current study comprised 2–354 participants who completed all three waves (50.3% females, SD = 1.77). Participants’ ages at T1 ranged from 6 years to 14 years (Mage = 9.62).

Both university and administrative departments at the data collection schools were approved by the Research Ethics Committee to conduct the research (approval no. K2020025 and KS2022913). The study’s purpose, process, participants’ rights, privacy protection, and data-retention policy were introduced to all voluntary participants. Consent was provided by participants, and the legal guardians permitted their participation in the study.

### 2.2 Measures

PTSS were measured using the Chinese version of the 13-item Child Revised Impact of Events Scale (38). Participants rated each item on a six-point frequency scale (0 = not at all; 5 = often). The total scores ranged from 0 to 65, with a higher score representing a higher level of PTSD symptoms. The scale has been shown to have good validity and reliability in previous studies (39). The scale showed good internal consistency in the current study. Cronbach’s alpha coefficients for PTSS at T1, T2, and T3 were 0.89, 0.87, and 0.87, respectively.

IA was measured using the Chinese version of the 20-item Young’s Internet Addiction Test (40). A five-point Likert scale was used to rate each item (1 = does not apply; 5 = always), with total scores ranging from 20 to 100. A higher total score indicates a more severe IA level. A prior study demonstrated the test’s great validity and reliability (41). The scale showed excellent internal consistency in the current study. Cronbach’s alpha coefficients for IA at T1, T2, and T3 were 0.93, 0.94, and 0.95, respectively.

LS was measured using the Chinese version of the five-item Satisfaction with Life Scale (20). Each item is rated on a six-point scale from 1 (strongly disagree) to 6 (strongly agree), with the overall score lying between 5 and 30. A higher total score represents a higher level of LS. The robust validity and reliability of this scale was demonstrated by a previous study (42). In this study, the scale showed an acceptable internal consistency. Cronbach’s alpha coefficients for LS at T1, T2, and T3 were 0.77, 0.83, and 0.86, respectively.

### 2.3 Statistical analysis

First, a descriptive analysis of all the study variables was conducted and a correlation matrix constructed using SPSS (Version 24). Second, a longitudinal mediation approach was applied using structural equation modeling (SEM) to examine the relationships among PTSS, IA, and LS across the three waves. This method allowed all three variables to be included in the model at each time point and enabled the analysis of all possible mediation pathways (43). It provides a robust framework for assessing whether the effect of a predictor on an outcome is mediated by variables across different time points while accounting for potential confounding factors (43). The study variables (i.e., PTSS, IA, and LS across the three waves) were modeled as latent variables, with subscales for each item as indicators. Age and sex were used as covariates that might be linked with the study variables. Furthermore, tests for common-method bias, temporal differences, measurement equivalence, and univariate normality were conducted across the three waves. AMOS (Version 23) was employed to assess the significance of mediation effects using 5–000 bootstrapped replications. Maximum likelihood estimation was applied to assess the parameters. Some suggested indexes and their standards were used to estimate the goodness-of-fit and quality-of-mediation model (44). An acceptable model required that the ratio of χ^2^ to degree of freedom should be smaller than 5.0. The Tucker-Lewis (TLI), normed fit (NFI), and comparative fit (CFI) indices should be higher than 0.90. The standard root mean square residual (SRMR) and root mean square error of approximation (RMSEA) should be lower than 0.08 and 0.05, respectively (44). As all participants consented to completing all the items, and no data were missing across all three waves.

## 3 Results

### 3.1 Descriptive findings

The descriptive-analysis results, including means, standard deviations, and intercorrelations among the study variables, are presented in **Table 1**. Significant correlations were observed among IA, LS, and PTSS across the three study waves; however, T3 PTSS did not significantly correlate with T1 IA (r = .035, *p* = .086), T1 LS (r = –.023, *p* = .275), and T2 LS (r = –.023, *p* = .262). All study variables were significantly intercorrelated after controlling for sex and age, excluding a nonsignificant intercorrelation between T1 LS and T3 PTSS (r = –.038, *p* = .066).

**Table 1:** Means, standard deviations, and correlations among study variables (n = 2354).

Variables	M±SD	1	2	3	4	5	6	7	8	9
1 T1 IA	31.74±13.71	/	-.313***	.253***	.468***	-.229***	.164***	.173***	-.085***	.058**
2 T1 LS	22.90±5.72	-.330***	/	-.182***	-.260***	.391***	-.127***	-.065**	.113***	-.038
3 T1 PTSS	14.28±14.52	.243***	-.178***	/	.155***	-.123***	.280***	.092***	-.058**	.119***
4 T2 IA	32.97±14.21	.508***	-.293***	.140***	/	-.343***	.255***	.213***	-.151***	.084***
5 T2 LS	23.03±5.81	-.262***	.412***	-.116***	-.394***	/	-.151***	-.119***	.215***	-.048*
6 T2 PTSS	13.84±12.96	.144***	-.119***	.279***	.217***	-.137***	/	.075***	-.075***	.160***
7 T3 IA	33.77±14.48	.230***	-.104***	.085***	.300***	-.178***	.056**	/	-.339***	.293***
8 T3 LS	23.29**±**5.94	-.114***	.134***	-.055**	-.197***	.246***	-.067**	-.366***	/	-.256***
9 T3 PTSS	11.24±11.51	.035	-.023	.119***	.042*	-.023	.163***	.249***	-.235***	/

[1] The left/bottom triangle displays the Pearson’s correlations of all study variables, and the right/top triangle displays the partial correlations of all the study variables after controlling for sex and age; [2] PTSS, post-traumatic stress symptom; IA, Internet addiction; LS, life satisfaction; [3] ***p < 0.001; **p < 0.01; and *p < 0.05.

Univariate normality was assessed by examining skewness and kurtosis values for all the study variables across the three waves (44). For LS, skewness ranged from –0.930 to –0.822 and kurtosis from 0.117 to 0.423. For IA, skewness ranged from 1.384 to 1.668 and kurtosis from 1.574 to 2.943. For PTSS, skewness ranged from 1.239 to 1.576 and kurtosis from 1.373 to 2.884. All values fell within the commonly accepted thresholds for approximate normality (i.e., |skewness| < 2 and |kurtosis| < 7), indicating that the variables exhibited acceptable levels of univariate normality (44).

To assess measurement equivalence across the three waves, a series of increasingly restrictive confirmatory-factor-analysis (CFA) models were conducted using AMOS (44). Criteria for establishing measurement invariance included the ΔCFI ≤ 0.01 and ΔRMSEA ≤ 0.015 (44). The configural invariance model (Model 1), which allowed all parameters to vary across time, demonstrated an acceptable fit (χ²/df = 2.935, CFI = 0.977, RMSEA = 0.029), indicating that the factor structure was consistent across waves. The metric invariance model (Model 2), which constrained factor loadings to be equal over time, also fit the data well (ΔCFI = 0.004, ΔRMSEA = 0.002), suggesting loading invariance. The scalar invariance model (Model 3), which added equality constraints on item intercepts, resulted in minimal change in fit relative to the metric model (ΔCFI = 0.004, ΔRMSEA = 0.001), supporting scalar invariance. Finally, the residual invariance model (Model 4), which further constrained item residual variances, showed a small but acceptable decline in fit (ΔCFI = 0.012, ΔRMSEA = 0.005), indicating partial support for strict invariance. Overall, these findings provide evidence for longitudinal measurement invariance, supporting meaningful comparisons of the latent constructs across waves.

Harman’s one-factor CFA model showed poor fit to the data (CFI = 0.322, NFI = 0.319, TLI = 0.285, SRMR = 0.141, RMSEA = 0.150), indicating that common-method bias was unlikely to be a serious concern in this study. Moreover, repeated-measures analysis of variance was used to test of temporal differences in the study variables. PTSS showed a highly significant change over time (F = 45.90, *p* <.001), indicating substantial variation across the three waves. IA also demonstrated a significant time effect (F = 18.72, *p* <.001), although the magnitude of change was smaller than that of PTSS. LS showed a modest but still statistically significant change over time (F = 3.63, *p* <.05). Overall, these results support the use of a longitudinal mediation model for further analysis using SEM.

### 3.2 The complete longitudinal mediation model

**Figure 1:**
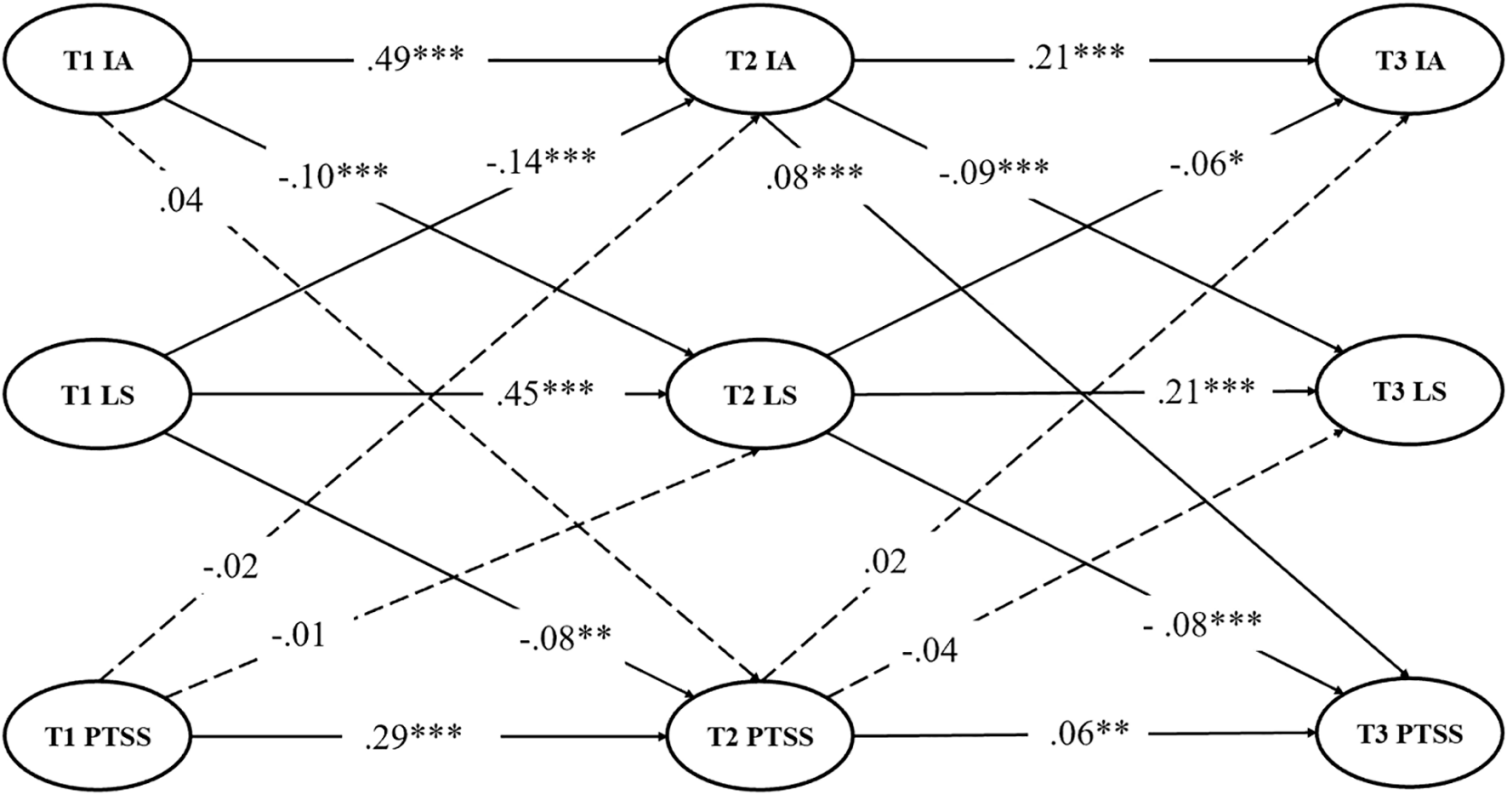
Complete longitudinal mediation model with standardized path coefficients. Dashed lines show insignificant paths. For conciseness, controlling variables (i.e., age and sex) and their factor loadings are not shown for the latent variables. IA, Internet addiction, LS, life satisfaction, PTSS, post-traumatic stress symptoms. ***p < 0.001, **p < 0.01, and *p < 0.05.

A complete longitudinal mediation model was estimated to evaluate the links among PTSS, IA, and LS across the three waves by controlling for sex and age as covariates (see **Figure 1**). The model demonstrated good fit (χ^2^/df = 4.629, CFI = 0.952, NFI = 0.940, TLI = 0.947, SRMR = 0.058, RMSEA (90% CI) = 0.039 (0.038–0.041)).

Results from the complete longitudinal mediation model revealed several significant pathways among the study variables across the three waves. Specifically, T1 IA positively predicted T2 IA (β = 0.492, SE = 0.031, *p* <.001) and negatively predicted T2 LS (β = –0.103, SE = 0.011, *p* <.001). T1 LS negatively predicted T2 IA (β = –0.142, SE = 0.146, *p* <.001) and positively predicted both T2 LS (β = 0.450, SE = 0.091, *p* <.001) and T2 PTSS (β = –0.081, SE = 0.214, *p* <.005). T1 PTSS positively predicted T2 PTSS (β = 0.285, SE = 0.022, *p* <.001). At the second wave, T2 IA positively predicted T3 IA (β = 0.205, SE = 0.024, *p* <.001), negatively predicted T3 LS (β = –0.087, SE = 0.009, *p* <.001), and positively predicted T3 PTSS (β = 0.083, SE = 0.016, *p* <.001). T2 LS negatively predicted T3 IA (β = –0.064, SE = 0.065, *p* <.001) and both positively predicted T3 LS (β = 0.213, SE = 0.026, *p* <.001) and negatively predicted T3 PTSS (β = –0.081, SE = 0.043, *p* <.001). T2 PTSS positively predicted T3 PTSS (β = 0.058, SE = 0.011, *p* <.005). Furthermore, results indicated some nonsignificant paths, including T1 IA to T2 PTSS (β = 0.043, SE = 0.046, *p* >.05), T1 PTSS to T2 IA (β = -0.016, SE = 0.013, *p* >.05) and T2 LS (β = -0.005, SE = 0.005, *p* >.05), and T2 PTSS to T3 IA (β = 0.020, SE = 0.017, *p* >.05) and T3 LS (β = -0.039, SE = 0.007, *p* >.05).

### 3.3 The clipped longitudinal mediation model

**Figure 2:**
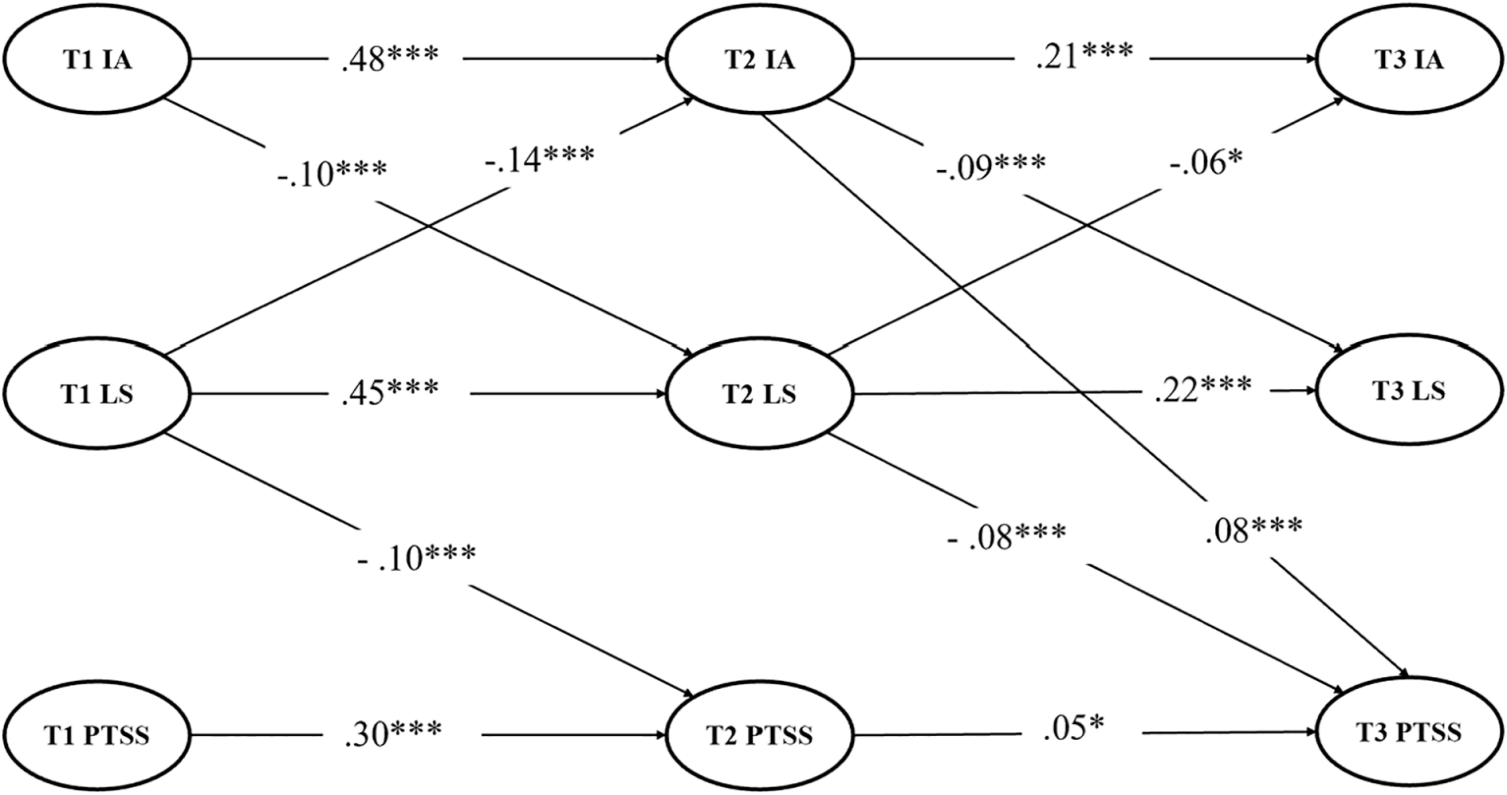
Clipped longitudinal mediation model with standardized path coefficients. Insignificant paths were removed in the model. For conciseness, controlling variables (i.e., age and sex) and their factor loadings are not shown for the latent variables. IA, Internet addiction; LS, life satisfaction; PTSS, post-traumatic stress symptoms. ***p <0.001, and *p < 0.05.

To enhance model conciseness, all nonsignificant paths were removed from the complete longitudinal mediation model, resulting in the clipped model (see **Figure 2**). The clipped model demonstrated a good goodness-of-fit (*χ^2^/df* = 4.587, CFI = 0.952, NFI = 0.939, TLI = 0.947, SRMR = 0.058, RMSEA (90% CI) = 0.039 (0.038–0.040)). The results revealed several significant relationships among IA, LS, and PTSS at adjacent time points. T1 IA significantly predicted T2 IA (β = 0.483, SE = 0.030, *p* <.001) and negatively predicted T2 LS (β = –0.10, SE = .010, *p* <.001). T1 LS negatively predicted T2 IA (β = –0.143, SE = 0.144, *p* <.001), positively predicted T2 LS (β = 0.453, SE = 0.092, *p* <.001), and negatively predicted T2 PTSS (β = -0.098, SE = 0.196, *p* <.001). T1 PTSS significantly predicted T2 PTSS (β = 0.296, SE = .021, *p* <.001). At the next time point, T2 IA significantly predicted T3 IA (β = 0.211, SE = .023, *p* <.001), negatively predicted T3 LLS (β = –0.094, SE = 0.009, *p* <.001), and positively predicted T3 PTSS (β = 0.081, SE = 0.015, *p* <.001). T2 LS negatively predicted T3 IA (β = –0.061, SE = 0.065, *p* = .013), positively predicted T3 LS (β = 0.218, SE = 0.026, *p* <.001), and negatively predicted T3 PTSS (β = –0.08, SE = 0.043, *p* <.001). T2 PTSS significantly predicted T3 PTSS (β = 0.045, SE = 0.009, *p* = .011). Overall, the results of the complete longitudinal mediation (**Figure 1**) and clipped (**Figure 2**) models revealed largely consistent findings regarding the relationships among the study variables across the three waves. Both models supported the same overall pattern of associations and underlying structure.

The results showed that T2 LS mediated the link between T1 IA and T3 PTSS [indirect effect (95% CI) = 0.047 (0.027–0.069)] and T2 IA mediated the relationship between T1 LS and T3 PTSS [indirect effect (95% CI) = –0.052 (–0.074 to –0.035)]. T2 LS mediated the association between T1 IA and T3 IA [indirect effect (95% CI) = 0.108 (0.080–0.141)]. T2 IA mediated the relationship between T1 LS and T3 LS [indirect effect (95% CI) = 0.112 (0.085–0.139)].

The results revealed consistent and significant bidirectional relationships among IA, LS, and PTSS across the three waves in the longitudinal mediation model. Specifically, at T1, LS was negatively correlated with both IA (r = –.445, p <.001) and PTSS (r = –.228, p <.001), while IA was positively correlated with PTSS (r = .272, p <.001). At T2, LS remained negatively correlated with IA (r = –.326, p <.001) and PTSS (r = –.133, p <.001), and IA was positively correlated with PTSS (r = .224, p <.001). Similarly, at T3, LS was negatively correlated with IA (r = –.349, p <.001) and PTSS (r = –.329, p <.001) while IA was positively correlated with PTSS (r = .321, p <.001). These findings demonstrate stable and reciprocal associations among the three study variables over time.

## 4 Discussion

This study employed a longitudinal mediation model to elucidate the relationships among PTSS, IA, and LS in a three-wave sample of Chinese children and young adolescents exposed to a major disaster. Our findings provided robust and large-sample evidence supporting the critical mediating role of LS as a potential mechanism linking early IA to subsequent PTSS in this population. Moreover, the results revealed that early LS significantly influenced prolonged PTSS through the mediating effect of IA following disaster exposure.

### 4.1 LS as a mediator between IA and PTSS

The results revealed that LS at the one-year follow-up significantly mediated the relationship between baseline IA and PTSS at the two-year follow-up. This finding supports the hypothesis and aligns with previous applications of self-determination theory (29, 30) and CBM (32, 33) in the context of IA. Specifically, higher IA levels in the early stages of a disaster may lead to reduced LS, which in turn increases the long-term risk of PTSS among Chinese children and early adolescents exposed to the disaster. This is consistent with prior research indicating an upward trend in IA among youth following traumatic events (4). Individuals with IA often experience discomfort and negative life changes, which may reduce their LS after a disaster (18). Lower LS can contribute to dissatisfaction with real life, deficits in quality of life, impaired social relationships, and disrupted life habits, ultimately heightening vulnerability to psychological problems (17). This result may also be explained by previous research showing that a higher degree of IA can impair individuals’ perceptions of LS derived from real-life social networks (45–47), thereby increasing the likelihood of long-term mental-health issues such as PTSS (48–50).

### 4.2 IA as a mediator between LS and PTSS

Interestingly, the findings revealed that the IA level at the one-year follow-up significantly mediated the relationship between baseline LS and PTSS at the two-year follow-up. This suggests that lower LS at the onset of a disaster might contribute to increased IA over time, which in turn heightens the risk of long-term psychological problems such as PTSS. Previous research has shown that disaster events such as the COVID-19 pandemic often disrupt daily life through factors such as social isolation, repeated testing, and regional lockdowns, all of which contribute to reduced LS (51–53). Furthermore, in the aftermath of a disaster, diminished LS may impair emotional regulation and increase vulnerability to stress, making individuals more likely to turn to the Internet as a form of avoidant coping (54). People with growing IA often rely on excessive online engagement to escape or numb distressing emotions in response to mounting real-life stressors (55–57). However, such avoidance hinders the emotional processing of trauma. When trauma remains unprocessed, it can become more deeply entrenched, leading to more severe PTSS (e.g., emotional numbing, intrusive thoughts, and hyperarousal) (58) following disasters. Additionally, although IA is frequently conceptualized as a mental-health outcome, our findings indicate that it could also function as a risk factor and mediator in the development of PTSS. This dual role aligns with prior evidence supporting the bidirectional relationships between IA and other mental-health problems (59, 60). Future research is suggested to further examine the underlying pathways through which IA longitudinally shapes psychological well-being in this dual role.

### 4.3 Bidirectional and mediating links involving PTSS, IA, and LS

Beyond the identified mediation pathways, the results revealed significant bidirectional associations between IA and PTSS across the three waves. These findings underscore the persistent, reciprocal relationship between IA and PTSS over time and are consistent with existing evidence highlighting strong intersections and potential comorbidity between the two [e.g (4, 14, 15).,]. Notably, no significant directed longitudinal effects were found from baseline PTSS to later LS or IA. One plausible explanation is that, within the framework of a two-year follow-up period, early PTSS may not exert a lasting impact sufficiently strong to influence long-term trajectories of subjective well-being or behavioral coping strategies, such as patterns of Internet use. Furthermore, children and early adolescents may possess greater psychological adaptability and benefit from external regulatory support (e.g., parental guidance), particularly during extended periods of home confinement and increased family time during the COVID-19 pandemic. Such support may buffer the enduring effects of trauma on daily functioning and LS (52). Additionally, both LS and Internet-use behaviors are shaped by a constellation of contextual factors, including peer relationships, academic pressures, and school dynamics, which may weaken the influence of PTSS over time (61–63). Collectively, these findings suggest that the longitudinal influence of PTSS on LS and IA may be governed by more complex and indirect mechanisms. Future research would benefit from examining potential mediators and moderators, such as personality traits, coping strategies, and family-relationship quality to further clarify these pathways (52, 64).

### 4.4 Strengths and limitations

Based on the large sample of disaster-exposed children and young adolescents and established measures, this three-wave study explored the associations among IA, LS, and PTSS from the perspective of a longitudinal mediation model. The different roles of LS in the mediating relationships indicated that IA and PTSS were closely linked but had independent patterns. These findings provide further evidence for the longitudinal mediated association mechanism between IA, LS, and PTSS. Notwithstanding these strengths, several limitations in the current study should be noted. First, the self-report questionnaire and assessment scales were used to evaluate the variables; this may lead to biased estimation because of social desirability, recall partiality, misinterpretation, or lack of insight (65). Future research may employ clinical diagnostic interviews (e.g., CAPS-5) or multiple-informant reports to improve measurement accuracy (66). Second, this study used a three-wave longitudinal design, which clarified temporal order and allowed tests of longitudinal mediation among IA, LS, and PTSS. The findings remain longitudinally correlational and predictive. However, since the longitudinal observational study was limited in the experimental conditions, such as manipulation and randomization, future experimental or intervention studies are needed to provide more conclusive causal evidence (67). Finally, these findings may not be generalizable to other disaster-exposed populations or to individuals who have experienced different types of trauma. Future research is recommended to investigate the causal relationships among the study variables across diverse populations and trauma exposures.

### 4.5 Practical implications

The present findings emphasize the protective role of LS in buffering the long-term negative influence of IA and PTSS, the potentially adverse effect of inchoate IA symptoms on LS, and their impact on long-term psychological health among children and young adolescents exposed to disasters. These results highlight the need for developing psychological intervention programs that strengthen positive life forces, enhance social support, and promote adaptive coping strategies in disaster-affected youth (68, 69). Customized interventions focusing on LS improvement may increase resilience and reduce excessive reliance on the virtual world following traumatic experiences. For example, mindfulness-based interventions (70), solution-focused brief therapy (71), and positive psychology interventions (72) have shown promise in boosting positive emotions, meaning, and LS in the aftermath of disasters. Policymakers and practitioners should consider incorporating these strategies into pre- or post-disaster support systems for vulnerable children and young people.

### 4.6 Conclusions

This exploratory study revealed that life satisfaction plays a vitally protective role by mediating the influence of early Internet addiction on long-term psychological outcomes. Moreover, Internet addiction mediated the links between earlier life satisfaction and later PTSS, indicating that both enhancing life satisfaction and reducing problematic Internet use are critical for mitigating mental symptoms among disaster-exposed youth. Future research should investigate these pathways by using longer-term dynamic tracking and multi-method approaches to elucidate causal mechanisms and aid the development of targeted interventions.

## Data Availability

The raw data supporting the conclusions of this article will be made available by the authors, without undue reservation.
